# Intensive animal production as driver of biodiversity loss and pandemics

**DOI:** 10.1093/eurpub/ckac129.020

**Published:** 2022-10-25

**Authors:** S Viegas

**Affiliations:** 1 Health & Technology Research Center, Lisbon, Portugal; 2 Escola Superior de Tecnologia da Saúd, Lisbon, Portugal

## Abstract

Pandemics have their origin in diverse microbes carried by animal hosts, but their emergence is entirely driven by human activities. These include deforestation, land- and sea-use change, agricultural expansion and intensification, and wildlife trade and consumption. These activities bring wildlife, livestock, and people into closer contact, allowing animal microbes to spillover into people and causing infections, sometimes outbreaks, and more rarely epidemics and pandemics. Domestic animals and peri-domestic wildlife also have a role in creating bridges for the emergence of human diseases, since this can happen in an evolutionary sense, or the animal could serve as a physical transmitter. The most important reservoirs of pathogens with pandemic potential are mammals (in particular bats, rodents and primates) and some birds, as well as livestock (e.g. pigs, mink, poultry). In fact, intensive animal production is also considered one of the drivers for biodiversity loss and potentially for future pandemics. As an example, intensive poultry farming not only poses a significant risk to workers, but can also act as a potential public health menace evidencing the One health approach to tackle all the menaces in this particular setting.

